# Epi-DNNs: Epidemiological priors informed deep neural networks for modeling COVID-19 dynamics

**DOI:** 10.1016/j.compbiomed.2023.106693

**Published:** 2023-05

**Authors:** Xiao Ning, Linlin Jia, Yongyue Wei, Xi-An Li, Feng Chen

**Affiliations:** a State Key Laboratory of Bioelectronics, School of Biological Science and Medical Engineering, Southeast University, 2 Sipailou, Nanjing, 210096, PR China; b Center for Global Health, Departments of Epidemiology and Biostatistics, School of Public Health, Nanjing Medical University, Address Two, Nanjing, 21166, PR China; c The COBRA Lab, INSA Rouen Normandie, 1 Rue Tesniere, Mont-Saint-Aignan, 76821, France; d Ceyear Technologies Co., Ltd, 98 Xiangjiang Road, Qingdao, 266000, PR China; e Public Health and Epidemic Preparedness and Response Center, Peking University, Xueyuan Road, Haidian District, Beijing, 100191, PR China

**Keywords:** 34A34, 68T07, Compartmental models, Deep neural networks, Parameter estimation, Runge–Kutta method, COVID-19

## Abstract

Differential equations-based epidemic compartmental models and deep neural networks-based artificial intelligence (AI) models are powerful tools for analyzing and fighting the transmission of COVID-19. However, the capability of compartmental models is limited by the challenges of parameter estimation, while AI models fail to discover the evolutionary pattern of COVID-19 and lack explainability. This paper aims to provide a novel method (called Epi-DNNs) by integrating compartmental models and deep neural networks (DNNs) to model the complex dynamics of COVID-19. In the proposed Epi-DNNs method, the neural network is designed to express the unknown parameters in the compartmental model and the Runge–Kutta method is implemented to solve the ordinary differential equations (ODEs) so as to give the values of the ODEs at a given time. Specifically, the discrepancy between predictions and observations is incorporated into the loss function, then the defined loss is minimized and applied to identify the best-fitted parameters governing the compartmental model. Furthermore, we verify the performance of Epi-DNNs on the real-world reported COVID-19 data on the Omicron epidemic in Shanghai covering February 25 to May 27, 2022. The experimental findings on the synthesized data have revealed its effectiveness in COVID-19 transmission modeling. Moreover, the inferred parameters from the proposed Epi-DNNs method yield a predictive compartmental model, which can serve to forecast future dynamics.

## Highlights

• Compartmental models with time-varying parameters capture COVID-19 dynamics well.
• Modeling COVID-19 dynamics by coupling the compartmental model and neural networks.
• Using neural networks to express time-varying parameters in compartmental models.
• Applying Fourier transformation to reduce the stochastic and noise of real-world data.
• Analyzing the effect of intervention policies and providing predictions.

## 1 Introduction

Epidemic compartmental models categorized the population into different compartments to analyze the transmission dynamics of infectious diseases based on disease status, serving as an incredibly powerful tool for detecting, understanding, and combating outbreaks [1]. Kermack and McKendrick first constructed a fundamental susceptible–infected–recovered (SIR) compartmental model to study the transmission dynamics of the Black Death in London, the United Kingdom in the year 1927 [2]. Since the start of COVID-19, SIR compartmental model and its variants Susceptible–Exposed–Infectious–Removed (SEIR) [3], Susceptible–Infected–Recovered–Death (SIRD) [4], [5], [6], Susceptible– Exposed–Infectious–Quarantine–Removed (SEIQR) [7], Susceptible– Exposed–Infectious–Hospitalized–Removed (SEIHR) [8], et al. have been at the forefront of studying the transmission dynamics of COVID-19 outbreak and the impact of various non-pharmaceutical interventions. These compartmental models are formulated by a system of ordinary differential equations (ODEs), which are characterized by a set of parameters that are not known prior and required to be identified from data. Therefore, parameter estimation methods are frequently required to compute the parameters of the flow from one compartment to another in these compartmental models. In addition, compartmental models assume constant parameters of the flow from one compartment to another to reduce the complexity of modeling. Many research efforts focus on parameter estimation of compartmental models using maximum likelihood estimation, Markov Chain Monte Carlo (MCMC)-based Bayesian inference [9], [10], [11], and finite element methods [12]. However, these methods suffer from significant limitations which hinder their applications. One limit is that the computational cost of numerical simulations increases exponentially with the complexity of the parameters and models. Another limitation is that these parameter estimation methods are only suited for time-constant parameters, which fail to reflect the complex dynamic of the infectious disease over time in real-world scenarios.

Artificial intelligence(AI), especially deep neural networks (DNNs) models also played an important role in analyzing and fighting the transmission of the COVID-19 epidemic [13], [14]. Despite AI models having great power to fit the data and provide short-time prediction, two main weaknesses hinder their practical applications. One is that they cannot find the patterns of the disease transmission process and will suggest not reasonable predictions due to ignoring biological reality. In addition, they depend heavily on the quality and quantity of data, the model may not be useful if the data do not reasonably capture reality. Compartmental models and AI models have both been shown to be reliable tools in fighting against the COVID-19 pandemic, along with their corresponding limitations, respectively. Therefore, exploring how to combine compartmental models and AI models to enhance their performance is a promising research topic. Recently, Physics-informed Neural Networks (PINNs) approaches have shown success in combining differential equations into the neural networks to satisfy the equations while accurately fitting the data [15]. That is, using neural networks to model nonlinear systems, but reducing the required data and constraining the model’s search space with prior knowledge such as a system of differential equations. Since then, DNNs-based models are consistently used as the non-linear function approximation method and have shown their strong potential to address various science computing tasks in many fields. Additionally, several research efforts have attempted to apply the PINNs framework in modeling and analyzing the dynamics of COVID-19 [16], [17], [18], [19], [20].

The concept of PINNs was first proposed for time-dependent partial differential equations (PDEs), which provide a flexible computational framework to address various science computing tasks. Inspired by PINNs, we proposed an Epi-DNNs method to model the complex outbreak dynamics of COVID-19 by integrating real-world data, epidemic transmission laws, and numerical ODE solvers into DNNs. Specifically, we build DNNs to express the unknown parameters in the compartmental model and introduce a numerical ODE solver to solve the corresponding ODEs so as to give the values of the ODEs at a given time. The discrepancy between predictions and observations is formulated as the loss function, which is minimized and applied to identify the best-fitted parameters governing the compartmental model. We verify the effectiveness of the proposed Epi-DNNs method on the COVID-19 reported data in the real world across several regions. The findings of simulation experiments demonstrated that the proposed Epi-DNNs robustly perform data-driven parameter estimation for the COVID-19 transmission modeling. Thus, the main contributions of this paper are as follows:

• To efficiently respond to the complexity of infectious disease transmission dynamics in the real world, we propose a method that combines mathematical modeling and neural network modeling. The proposed method considers the coefficients of the epidemic compartmental model as time-varying parameters that provide an accurate capture of transmission dynamics and reliable predictions.
• We build separate neural networks for each time-varying parameter in the epidemic compartmental model respectively and perform Fourier transformation for the input data to reduce the inherent stochastic and noisy nature of real-world data. The proposed method offers a feasible way to efficiently estimate time-varying parameters, instead of handling time-varying parameters by dividing them into different time intervals, as conventional parameter estimation methods are limited to.
• We apply the proposed method to real-world reported COVID-19 data to validate its effectiveness. More importantly, the proposed Epi-DNNs approach can be easily adapted to other compartmental models, providing a convenient way to model and analyze the dynamics of infectious disease transmission in any region.

The remaining of this paper is organized as follows: in Section 2, we briefly present the related works of COVID-19 transmission modeling. In Section 3, we introduce the Fourier-induced neural networks, the SIRD compartmental model, and the overview of the proposed Epi-DNNs method as well as its implementation details. In Section 4, we present simulation results based on the real-world reported data. Then, in Section 5, we present some discussions and suggestions. Finally, a brief conclusion is made in Section 6.

## 2 Related works

The coronavirus disease 2019 (COVID-19) and its related impact have emerged as one of the most complex and threatening public health challenges ever encountered. Given uncertainties in the transmission of COVID-19, and the impact of infections, hospitalizations, and deaths, the infectious disease transmission models have been widely used since the outbreak to answer a number of questions for decision-makers. Modeling approaches for studying infectious disease transmission primarily include compartmental models, statistical models, ensemble models, and individual models.

Compartmental models allow as much complexity in the model as is necessary and can represent non-linear processes and feedback. Li et al. applied a networked dynamic meta-population mode and Bayesian inference to infer the proportion of undetected individuals in COVID-19 early infections and analyze their contribution to virus spread [21]. Tian et al. performed a quantitative analysis of the effectiveness of control measures between December 31, 2019 and February 19, 2020, using a data set that included case reports, human movement, and public health interventions [22]. Wei et al. proposed an extended SEIR mode to evaluate how the implementation of clinical diagnostic criteria and universal symptom survey contributed to COVID-19 control in Wuhan, China [23]. Wang et al. used a modeling approach to reconstruct the full-spectrum dynamics of COVID-19 in Wuhan between January 1 to March 8, 2020 across 5 periods defined by events and interventions, identified the high covertness and high transmissibility features of the outbreak [24]. Liu et al. took the conversion rate between asymptomatic infections and reported/unreported symptomatic infections into account, and proposed an infectious dynamics model that adapts to all-people testing (APT). It adapted to densely populated metropolises for APT on prevention, where the result seemed more reasonable, and epidemic prediction became more accurate [25]. The primary limitation of these compartmental models is that they are subject to assumptions about the transmission process and the parameters.

Statistical models depend heavily on the quality and quantity of historical data used to make the prediction. Ensemble models are a compilation of multiple model outputs, which mitigate the risk of relying on just one model. Individual models incorporate each individual in the population as a separate agent in the model with their own individual assumptions and parameters. Each of these four categories of model structures has advantages and limitations. AI technologies have been intensively applied to modeling COVID-19, including daily infection prediction, medical imaging, health and clinic records, protein sequences, and drug discovery, et al. AI plays a significant role to control the COVID-19 pandemic disease, *Intelligent Systems and Methods to Combat Covid-19* collection [26] categorizes and summarizes different intelligent systems and methods to prevent further COVID-19 spreading and provides a detailed description of various application scenarios. Chimmula et al. applied the long short-term memory (LSTM) networks to model the spread of infectious diseases in Canada to predict the severity of COVID-19 [27]. Jayanthi et al. used the Auto-regressive Integrated Moving Average (ARIMA) model, LSTM, Stacked LSTM, and Prophet [28] models to analyze and predict the global cumulative number of confirmed cases, death cases, and recovered cases [29]. Nabi et al. studied four deep learning models: LSTM, Gated recurrent unit (GRU) networks, Convolutional neural networks (CNN), and Multivariate CNN to understand the future dynamics of COVID-19 flawlessly [30].

Physics-informed machine learning introduces a learning bias by directly embedding prior knowledge to make a more accurate and robust performance. Recent studies have successfully applied physics-informed machine learning to study the complex outbreak dynamics of COVID-19 by integrating advanced epidemiology models into deep neural networks. Kharazmi et al. analyze several epidemiological models through the lens of PINNs to identify time-dependent parameters and data-driven fractional differential operators [18]. Long et al. proposed a variant of PINNs to identify the time-varying parameters of the Susceptible–Infectious–Recovered–Deceased model for the spread of COVID-19 by fitting daily reported cases [20]. Nascimento et al. proposed an approach that can implement hybrid models combining physics-informed and data-driven kernels, where the latter are used to reduce the gap between predictions and observations [31]. Cai et al. adopted a Caputo–Hadamard fractional derivative to refine the classical susceptible–exposed–infected–removed model, then inferring the fractional order and time-dependent parameters as well as unobserved dynamics of the fractional SEIR model via fractional physics-informed neural networks [32].

## 3 Modeling methodology

### 3.1 Neural network modeling

#### Deep neural networks.

Mathematically, a deep neural network (DNN) defines a mapping of the form

$$F:x\in R^{d}⟹y=F\left(x\right)\in R^{c},$$

where d and c are the dimensions of the input and output, respectively. Generally, a standard neural unit of a DNN receives an input $x\in R^{d}$ and produces an output $y\in R^{m}$, i.e., y=σ(Wx+b) with $W\in R^{d\times m}$ and $b\in R^{m}$ being weight matrix and bias vector, respectively. σ(⋅), which is referred to as the activation function, is designed to add element-wise non-linearity to the model.

A DNN with L hidden layers can be regarded as a nested composition of sequential standard neural units. For convenience, we denote the output of the DNN by y(x;θ) with θ standing for the set of all weights and biases. Specifically, the $j_{th}$ neuron in ℓ layer can be formulated as

(1) $$y_{j}^{\left[\ell \right]}=\overset{N^{\left[\ell -1\right]}}{\underset{k=1}{\sum }}w_{jk}^{\left[\ell \right]}*\sigma^{\left[\ell -1\right]}\left(y_{k}^{\left[\ell -1\right]}\right)+b_{j}^{\left[\ell \right]},$$

where $y_{k}^{\left[\ell -1\right]}$ represents the value of the $k_{th}$ neuron in the ℓ−1 layer, $N^{\left[\ell -1\right]}$ represents the number of neurons in the ℓ−1 layer, $\sigma^{\left[\ell -1\right]}$ is the activation function of the ℓ−1 layer, $w_{jk}^{\left[\ell \right]}$ is the weight between the $k_{th}$ neuron in the ℓ−1 layer and the $j_{th}$ neuron in the ℓ layer, and $b_{j}^{\left[\ell \right]}$ is the bias of the $j_{th}$ neuron in the ℓ layer.

#### ResNet block.

**Fig. 1:**
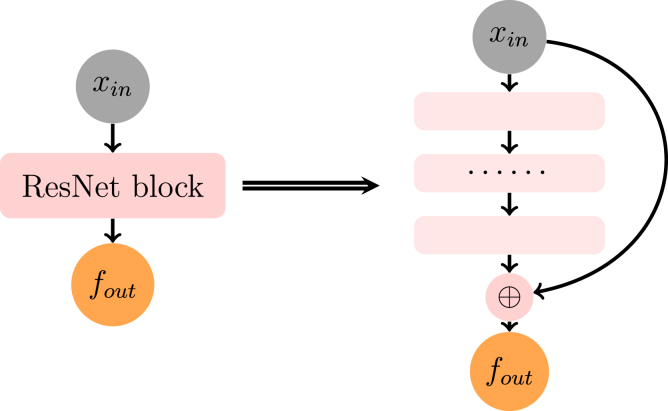
The architecture of ResNet.

Residual Network architecture (ResNet) was proposed to solve the problem of vanishing/exploding gradient of deep convolutional neural networks in computer vision tasks [33]. The key idea of ResNet is the skip connections by allowing alternate shortcut path for the gradient to flow through, which enables the model to learn the identity functions to guarantee that the higher layer will perform at least as good as the lower layer. For the given advantages, ResNet methods have been widely used in DNNs for solving PDEs and have shown extraordinary performance in approximating the solution and high-order derivatives of PDEs [34], [35]. The architecture of ResNet is depicted in Fig. 1, where a ResNet block with a one-step connection produces a filtered version $y^{\left[\ell +1\right]}\left(x;\theta \right)$ for the input $y^{\left[\ell \right]}\left(x;\theta \right)$ as follows

(2) $$y^{\left[\ell +1\right]}\left(x;\theta \right)=y^{\left[\ell \right]}\left(x;\theta \right)+\sigma \circ \left(W^{\left[\ell +1\right]}y^{\left[\ell \right]}\left(x;\theta \right)+b^{\left[\ell +1\right]}\right).$$

#### Fourier mapping.

The activation function is one of the critical factors for designing the architecture of DNNs. As a non-linear transformation that bounds the value of the input data, it directly affects the performance of DNNs models in practical applications. Several different types of activation functions have been used in DNNs, such as ReLU(z)=max{0,z} and tanh(z).

A well-proven phenomenon is that DNNs show a spectral bias or frequency preference, that is, DNNs will first capture the low-frequency components of input data [36]. Within this sense of spectral bias and Fourier approximation, a given real function F(x) can be expressed in the following sine and cosine expansions:

$$F\left(x\right)=\overset{\overset{\sim }{N}}{\underset{n=1}{\sum }}\left(S\left(\cos\left(\omega_{n}x\right);\overset{\sim }{\theta }\right)+T\left(\sin\left(\omega_{n}x\right);\overset{¯}{\theta }\right)\right),$$

where $S\left(x,\overset{\sim }{\theta }\right)$ and $T\left(x,\overset{¯}{\theta }\right)$ represent DNN modules, and $\left\{\omega_{0},\omega_{1},\omega_{2},\ldots \right\}$ are the frequencies of interest in the target function, where ω=0 will always be included.

**Fig. 2:**
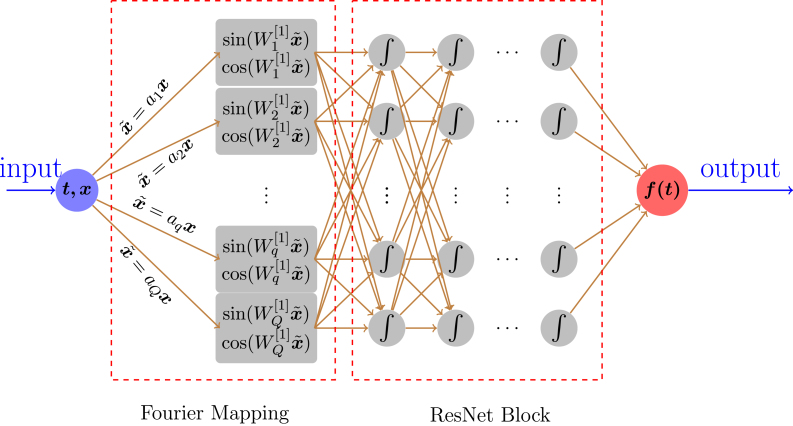
Illustration of the representation of Fourier basis in DNNs with ResNet block. $W_{q}^{\left[1\right]}$ represents the weight of the qth hidden units in the first hidden layer.

Definitely, recent works have shown that using Fourier feature mapping as an activation function can remarkably improve the capacity of DNNs [37], [38], [39], [40], [41]. Therefore, a novel activation function can be expressed as Eq. (3) based on spectral bias and Fourier approximation. It can mitigate the pathology of spectral bias and enable networks to well learn the target function [37], [38]:

(3) $$\sigma \left(z\right)=\left[\begin{array}{c} \cos\left(2\kappa \pi z\right)\\ \sin\left(2\kappa \pi z\right) \end{array}\right],$$

where κ is a user-specified vector (not trainable) which is consistent with the number of neural units in the first hidden layer for DNNs. By performing a Fourier feature mapping of the input data, the input points in $R^{d}$ can be mapped to the range [−1,1]. After that, the following layers of the neural network can process the feature information in Fourier space efficiently. The neural network architecture part of the Epi-DNNs method is shown in Fig. 2.

### 3.2 Compartmental models

Compartmental models enable the simulation of multi-state population transitions by incorporating domain knowledge and mathematical assumptions to characterize the dynamics of infectious diseases. Transmission dynamics models of infectious diseases are generally represented as the following non-linear dynamical system:

(4) $$\left\{\begin{array}{c} \begin{array}{cc} \frac{du\left(t\right)}{dt} & =f\left(t,u\left(t\right);\Xi \right)\\ u\left(t_{0}\right) & =u_{0} \end{array}\; \end{array}\right)$$

where $u\in R^{D}$ (typically D≫1) is the state variable, $t\in \left[t_{0},T\right]$ is the time, $u\left(t_{0}\right)$ is the initial state, and Ξ stands for the parameters of dynamical system.

The basic Susceptible–Infection–Recovered–Death (SIRD) model is extended on the SIR model, which describes the interaction of the virus with the host during transmission, and divides the population into 4 types: susceptible, infected, recovered, and deceased. The SIRD model can be described by the following ordinary differential equations:

(5) $$\left\{\begin{array}{c} \begin{array}{cc} \frac{dS\left(t\right)}{dt} & =-\beta \frac{S\left(t\right)I\left(t\right)}{N}\\ \frac{dI\left(t\right)}{dt} & =\beta \frac{S\left(t\right)I\left(t\right)}{N}-\gamma I\left(t\right)-\mu I\left(t\right)\\ \frac{dR\left(t\right)}{dt} & =\gamma I\left(t\right)\\ \frac{dD\left(t\right)}{dt} & =\mu I\left(t\right)\\ N & =S\left(t\right)+I\left(t\right)+R\left(t\right)+D\left(t\right) \end{array}\; \end{array}\right)$$

where *S(t)*, *I(t)*, *R(t)*, *D(t)* denote the number of susceptible, infected, recovered and deceased individuals over time, respectively. β≥0 represents the *transmission rate* of the disease. γ≥0 represents the *recovery rate*, which is the proportion of infected individuals that recover from the disease per unit of time. μ≥0 is the *death rate*. The model is initialized at some conventional $t=t_{0}$ with values $S\left(t_{0}\right)=S_{0}>0,I\left(t_{0}\right)=I_{0}>0,R\left(t_{0}\right)=R_{0}\geq 0$, and $D\left(t_{0}\right)=D_{0}\geq 0$. R(t)+D(t) denotes the removed individuals that are removed from the susceptible compartment due to death or immunization.

In the basic SIRD model, the three parameters of transmission rate β, recovery rate γ, and death rate μ are considered time-constant. However, the long duration of the pandemic, the associated interventions implemented by authorities, and/or mutations of the virus, et al. result in the parameters in the SIRD model changing over time. Accordingly, compartmental models require time-varying parameters to accurately and effectively model the dynamic of COVID-19 epidemiological attributes including time-varying infection, recovery, and mortality rate. The time-varying SIRD model takes the transmission rate β, recovery rate γ and death rate μ as functions of time: β(t),γ(t),μ(t), the re-written differential equations are as follows:

(6) $$\left\{\begin{array}{c} \begin{array}{cc} \frac{dS\left(t\right)}{dt} & =-\beta \left(t\right)\frac{S\left(t\right)I\left(t\right)}{N}\\ \frac{dI\left(t\right)}{dt} & =\beta \left(t\right)\frac{S\left(t\right)I\left(t\right)}{N}-\gamma \left(t\right)I\left(t\right)-\mu \left(t\right)I\left(t\right)\\ \frac{dR\left(t\right)}{dt} & =\gamma \left(t\right)I\left(t\right)\\ \frac{dD\left(t\right)}{dt} & =\mu \left(t\right)I\left(t\right)\\ N & =S\left(t\right)+I\left(t\right)+R\left(t\right)+D\left(t\right) \end{array}\; \end{array}\right)$$

Among them, the four variables *S(t)*, *I(t)*, *R(t)*, and *D(t)* have the same meaning as in Eq. (6). If we assume that the total population N is constant, then the sum of the increase or decrease of the state of each population is 0, namely, $\frac{dS\left(t\right)}{dt}+\frac{dI\left(t\right)}{dt}+\frac{dR\left(t\right)}{dt}+\frac{dD\left(t\right)}{dt}=0$.

### 3.3 Overview of Epi-DNNs

Here, DNNs with input *t* are parameterized by a set of parameters as the hypothesis spaces (denoted as $Params_{NN}$) and implemented to represent the data-driven surrogate $Params_{NN}\left(\cdot ,\theta_{\beta },\theta_{\gamma },\theta_{\mu }\right)$. For given differential Eqs. (4), the task is to find the value of the unknown function f(t) at a given point t. Runge–Kutta method is an effective and widely used numerical method to solve differential equations, the numerical accuracy determined by its order. The classical fourth-order Runge–Kutta method is the most commonly used numerical ODE solver. The formula below is used to compute the next value f(u(t+1)) from the previous value f(u(t)). The value of t is $0,1,2,\ldots \left(t-t_{0}\right)/h$, where h is the step height and $t_{n+1}=t_{n}+h$.

(7) $$\left\{\begin{array}{c} \begin{array}{cc} K_{1} & =hf\left(t_{n},u_{n}\right)\\ K_{2} & =hf\left(t_{n}+\frac{h}{2},u_{n}+\frac{K_{1}}{2}\right)\\ K_{3} & =hf\left(t_{n}+\frac{h}{2},u_{n}+\frac{K_{2}}{2}\right)\\ K_{4} & =hf\left(t_{n}+h,u_{n}+K_{3}\right)\\ u\left(t+1\right) & =u\left(t\right)+\frac{K_{1}}{6}+\frac{K_{2}}{3}+\frac{K_{4}}{6}+O\left(\Delta t^{5}\right) \end{array}\; \end{array}\right)$$

The formula basically computes the next value $u_{t+1}$ using the current $u_{t}$ plus the weighted average of four increments. Then the expression of time-varying parameters β(t), γ(t), and μ(t) for the SIRD model can be obtained by minimizing the following loss function:

(8) $$Loss=\omega_{S}Loss2S+\omega_{I}Loss2I+\omega_{R}loss2R+\omega_{D}Loss2D+\omega \left(\theta_{\beta },\theta_{\mu },\theta_{\gamma }\right)$$

with

$$\left\{\begin{array}{c} Loss2S=\frac{1}{N}\overset{N-1}{\underset{n=0}{\sum }}|S_{n+1}-S_{n}-F{\left(S_{n},I_{n},Params_{NN}\left(t_{n}\right)\right)|}^{2}\;\\ Loss2I=\frac{1}{N}\overset{N-1}{\underset{n=0}{\sum }}|I_{n+1}-I_{n}-F{\left(S_{n},I_{n},Params_{NN}\left(t_{n}\right)\right)|}^{2}\;\\ Loss2R=\frac{1}{N}\overset{N-1}{\underset{n=0}{\sum }}|R_{n+1}-R_{n}-F{\left(I_{n},Params_{NN}\left(t_{n}\right)\right)|}^{2}\;\\ Loss2D=\frac{1}{N}\overset{N-1}{\underset{n=0}{\sum }}|D_{n+1}-D_{n}-F{\left(I_{n},Params_{NN}\left(t_{n}\right)\right)|}^{2},\; \end{array}\right)$$

where the observed data for S(t),I(t),R(t) and D(t) at $t=t_{0},t_{0}+1,t_{0}+2,\ldots$ with a given time interval $\left[t_{0},T\right]$ are denoted as $S_{0},S_{1},S_{2},\ldots$, $I_{0},I_{1},I_{2},\ldots$, $R_{0},R_{1},R_{2},\ldots$ and $D_{0},D_{1},D_{2},\ldots$, respectively. F stands for the nonlinear mapping of the fourth-order Runge–Kutta method for given input data and parameters. It should be noted that the fourth order Runge–Kutta Method has the local and global error of $O\left(\Delta t^{5}\right)$ and $O\left(\Delta t^{4}\right)$ with Δt being the step size, respectively. In addition, we introduce four positive relaxing factors $\omega_{I},\omega_{R},\omega_{D}$, and ω to balance the contribution of Loss2S, Loss2I, Loss2R, Loss2D and the regularization sum of network parameter in the loss function, respectively.

To obtain the ideal $\theta_{\beta }^{*},\theta_{\mu }^{*}$ and $\theta_{\gamma }^{*}$, optimization methods such as gradient descent (GD) or stochastic gradient descent (SGD) are required to update the parameters of the DNNs during the training. In this context, the SGD is given by:

$$\theta^{k+1}=\theta^{k}-\alpha_{k}\nabla_{\theta^{k}}L\left(t;\theta^{k}\right),\;\;t\in \left\{t_{0},t_{1},t_{2},\ldots \right\},$$

where the *learning rate* $\alpha_{k}$ decreases with k increasing and $\theta =\left\{\theta_{\beta },\theta_{\mu },\theta_{\gamma }\right\}$.

Algorithm 1 describes the workflow of the proposed Epi-DNNs method for solving nonlinear dynamical Eqs. (5).

**Figure:**
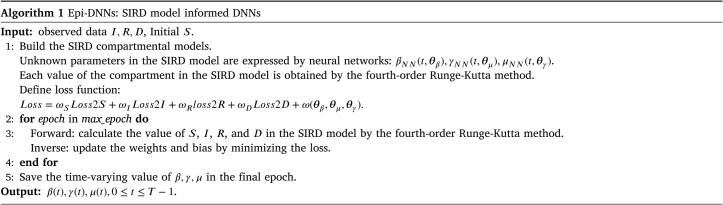

## 4 Numerical simulations

### 4.1 Experimental setting

#### Data.

The simulation is based on the real-world COVID-19 data announced on the official website by Shanghai Municipal Health Commission.^1^ The related data set includes exhaustive information on the time series I(t), R(t), and D(t) covering February 25 to May 27 2022. Here I(t) refers to the sum of confirmed and asymptomatic (here asymptomatic infections are assumed to transform into recovered status after 6 days [42]). These time series data are smoothed with a 7-day moving average to smooth out the errors in the data. Key events and corresponding dates are taken into account to better understand the evolution of the COVID-19 transmission in Shanghai, which are listed below:

• February 25, 2022: First asymptomatic case.
• **March 2, 2022**: Closure of partial public places.
• March 12, 2022: Closure of kindergartens, primary, secondary and vocational schools; limiting movement within city borders.
• March 14, 2022: Public transportation closures of the long- distance bus.
• March 15, 2022: Closure of universities; limiting gatherings.
• March 15–16, 2022: Lockdown and mass PCR screening within high-risk areas.
• March 18–20, 2022: Mass PCR screening within non-high-risk areas.
• March 23, 2022: Adoption of makeshift isolation hospitals; lockdown and mass PCR screening within high-risk areas.
• March 26–27, 2022: Lockdown and mass PCR screening within high-risk areas.
• **March 28, 2022**: Lockdown of eastern Shanghai.
• March 28–30, 2022: Mass PCR screening of eastern Shanghai.
• **April 1, 2022**: Lockdown of western Shanghai; mass PCR screening of western Shanghai.
• April 4, April 10, April 26, 2022: Mass PCR screening of all Shanghai.
• May 1–7, 2022: Mass PCR screening of all Shanghai.
• June 1, 2022: Lift lockdown.

#### Framework.

Each neural networks implemented in this paper comprise 5 layers, where the weight matrix $W_{k}$ and the bias vector $b_{k}$ of the kth layer are respectively $W_{1}\in R^{1\times 35}$, $W_{2}\in R^{35\times 50}$, $W_{3}\in R^{50\times 30}$, $W_{4}\in R^{30\times 30}$, $W_{5}\in R^{30\times 20}$ and $b_{1}\in R^{35}$, $b_{2}\in R^{50}$, $b_{3}\in R^{30}$, $b_{4}\in R^{30}$, $b_{5}\in R^{20}$. In this numerical experiment, all neural networks are trained by the Adam optimizer, where the initial learning rate is $2\times 10^{-3}$ with a decay rate 95% for every 1000 epochs. In addition, the regular factors ω is set as 0.0005, and max epoch is set as 100 000.

### 4.2 Result analyses

#### Data fitting.

**Fig. 3:**
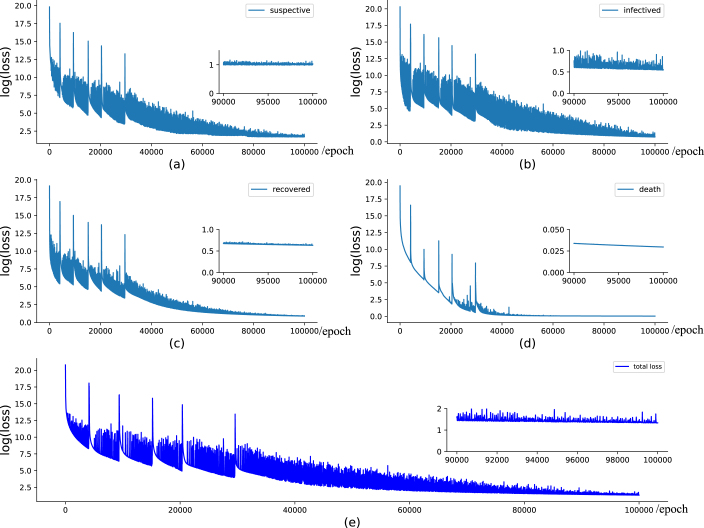
Data fitting performance: Loss of S, I, R, D and total loss during training. (a) Loss of S. (b) Loss of I. (c) Loss of R. (d) Loss of D. (e) Total loss.

We observe in Fig. 3 that the value of the loss function tends to decrease from the beginning to the end during the training process and gradually stabilizes in the range of minimal values. The formula of loss functions (8) indicates that it represents the differences between real-world data and predicted data, therefore well-performed loss function demonstrates the excellent fit between data and model.

#### Inferences.

**Fig. 4:**
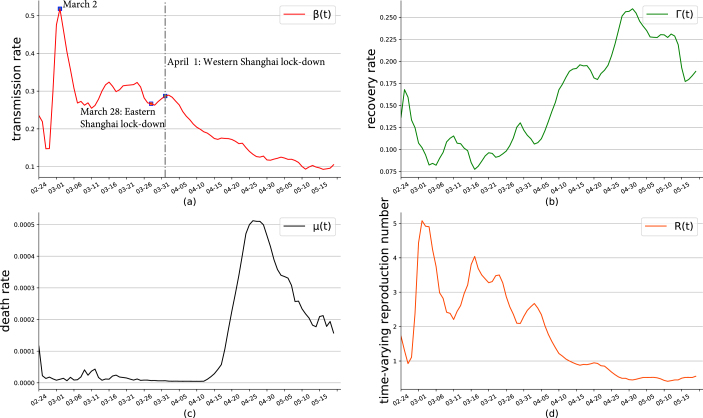
Epi-DNNs results: Inferences of model parameters based on the available data from February 25 to May 20 2022. (a) Transmission rate β(t). (b) Recovery rate γ(t). (c) Death rate μ(t). (d) Effective reproduction number R(t).

We are interested to infer the parameters β, γ, and μ by solving the inverse problem of the SIRD model. Fig. 4 shows the inferences of the time-varying parameters β(t), γ(t), and μ(t) from February 25 to May 20 2022. $R\left(t\right)=\frac{\beta \left(t\right)}{\gamma \left(t\right)+\mu \left(t\right)}$ represents the effective reproduction number, R(t) less than 1 indicates that the transmission of the infectious disease will gradually disappear. Further, observe that the time behavior of the fitted parameters is consistent with their expected dynamics. Since the high transmissibility and immune escape properties of the Omicron variant, the infections increased sharply following the first case. The authorities of Shanghai started imposing the closure of partial public places on March 2, followed by a series of interventions to combat the outbreak of Omicron. These interventions achieved a certain success, as demonstrated by a significant reduction in transmission rates and R(t). However, the outbreak was not under control (R(t)>1). On March 16, grid precise management was implemented but with limited effectiveness until March 26. As can be seen, the transmission rate behaves as a fluctuating oscillation, with R(t) consistently greater than 1. Until the lockdown of eastern Shanghai on March 28 and the lockdown of western Shanghai on April 1, transmission rates β(t) and effective reproduction number R(t) showed a continuous decreasing trend, with R(t) gradually approaching 0, and the outbreak was curbed. On the other hand, the recovery rate γ and the death rate μ are expected to increase and decrease, respectively, thanks to the use of more effective treatments for the disease.

#### Forecasting.

**Fig. 5:**
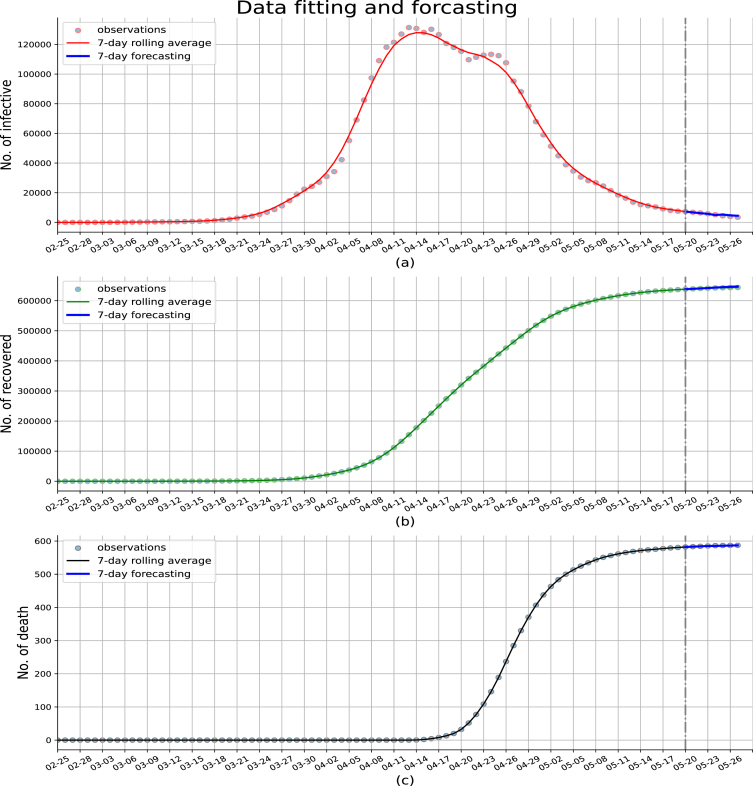
7-day predictions based on the time-varying SIRD model. The gray vertical line divides the fitting and prediction window. We have included the newly available data for the prediction period that was not used in the fitting to show the generalization ability of the model. (a) Current infections. (b) Cumulative recovery. (c) Cumulative deaths.

The non-linear ODEs system requires determined initial conditions and model parameters to make predictions. As the initial conditions can be obtained from the training data and the model parameters are already calibrated, we can forecast the epidemic dynamics by solving the forward problem. In the prediction part, the value of β(t), γ(t) and μ(t) are assumed to be their final value of the training time window. Fig. 5 depicts the data fitting and prediction obtained by using the identified time-varying model with the parameters given above. The perfect match between the predictions and the observations demonstrates the parameters inferred by the learned network are very plausible, as well as the generalization ability of the model.

### 4.3 Evaluation metrics

By comparing forecasting results and observations, the performance of the proposed Epi-DNNs can be evaluated. We use four evaluation metrics to make fair and effective comparisons. They are mean absolute error (MAE), average absolute percentage error (MAPE), root mean square error (RMSE), and relative error (REL). Their corresponding equations are shown in Eqs. (9) (10) (11), (12), respectively.

(9) $$MAE=\frac{1}{n}\overset{n}{\underset{i=1}{\sum }}\left|\overset{ˆ}{y_{i}}-y_{i}\right|,$$

(10) $$MAPE=\frac{1}{n}\overset{n}{\underset{i=1}{\sum }}\left(\frac{\overset{ˆ}{y_{i}}-y_{i}}{y_{i}}\right)*100\text{\%},$$

(11) $$RMSE=\sqrt{\frac{1}{n}\overset{n}{\underset{i=1}{\sum }}{\left(\overset{ˆ}{y_{i}}-y_{i}\right)}^{2}},$$

(12) $$REL=\overset{n}{\underset{i=1}{\sum }}\frac{{\left(\overset{ˆ}{y_{i}}-y_{i}\right)}^{2}}{{\overset{ˆ}{y_{i}}}^{2}},$$

To test the performance of the proposed Epi-DNNs method in the prediction, we did 3-days, 5-day, and 7-day experiments. The experimental results represented in Table 1 show the forecasting capability with high accuracy of the proposed Epi-DNNs method.

**Table 1:** The prediction performance in 3 days, 5 days and 7 days.

Metrics	After May 20, 2022		
	3-days	5-days	7-days
MAE of I	29.57	148.82	160.27
MAE of R	282.75	813.39	1495.28
MAE of D	0.07	0.38	0.39

MAPE of I	$1.22e^{-6}$	$6.14e^{-6}$	$6.61e^{-6}$
MAPE of R	$1.17e^{-5}$	$3.35e^{-5}$	$6.17e^{-5}$
MAPE of D	$2.87e^{-9}$	$1.58e^{-8}$	$1.60e^{-8}$

RMSE of I	39.16	222.27	213.63
RMSE of R	330.02	1079.20	1943.36
RMSE of D	0.09	0.55	0.53

REL of I	$3.95e^{-5}$	$1.50^{-3}$	$1.56^{-3}$
REL of R	$2.66e^{-7}$	$2.83e^{-6}$	$9.16^{-6}$
REL of D	$2.16e^{-8}$	$8.87e^{-7}$	$8.0^{-7}$

## 5 Discussion

The global pandemic COVID-19 has affected the lives of most people severely around the world, even causing numerous loss of lives. COVID-19 has reshaped the focus of global scientific attention and efforts, and researchers across the world have done much work to analyze the dynamic of COVID-19. Among them, exploring combining mathematical modeling and emerging AI technology to capture the complex outbreak dynamics of COVID-19 is a promising research topic. In this paper, we proposed the Epi-DNNs method to combine the deep learning method with the compartmental model to model the real-time dynamics of COVID-19. Experiment results demonstrate that the time-varying parameters of the compartmental model identified by the proposed Epi-DNNs method are consistent with expectations.

The transmission rate β determines the dynamics of the epidemic, and the time-varying β(t) estimated by the proposed Epi-DNNs method can accurately capture the changes in government interventions and individual behaviors. The recovery rate γ(t) and the death rate μ(t) are expected to increase and decrease, respectively, thanks to the more effective treatments for the disease. The identified γ(t) and μ(t) by our proposed Epi-DNNs method also fit well with the improved capacity of the healthcare system to fight against COVID-19. The effective reproduction number R is the transmission process of the virus, which represents the number of people transferred from the susceptible group to the infected group per unit of time. Chen et al. divided the epidemic into three phases to describe the epidemiological characteristics and spatiotemporal transmission dynamics of the Omicron outbreak in Shanghai and estimated the dynamics of R(t) [43]. Lou et al. constructed an extended compartmental model to retrospectively analyze the epidemic in Shanghai from 26 February 2022 to 31 May 2022 across four periods defined by related interventions and estimated R. As shown in Figure, the value of R(t) estimated by proposed Epi-DNNs method is consistent with those given by other researchers [44]. More importantly, by applying estimated parameters to the compartmental model to depict the dynamics of COVID-19, the perfect fitting between model predictions and observed data also underscores that parameters yield great fitness.

For different research scenarios, compartmental models are required to divide different compartments such as asymptomatic and symptomatic, adding the virus mutations, or adding the vaccination campaign. The proposed Epi-DNNs method is easy to be implemented without any background knowledge about numerical analysis (for example, stability conditions). For applying the Epi-DNNs method to other compartmental models, practitioners only needs to redefine the transformation matrix for each compartment according to the equations and build DNNs with the help of some libraries that implement deep neural networks. Therefore, the proposed Epi-DNNs method is applicable for parameter estimation of other compartmental models and other areas around the world and future infectious diseases. Although the proposed Epi-DNNs provide an important modeling method for infectious disease transmission, there are also some limitations. Due to that it is impossible to build a state-of-the-art compartmental model that can represent all the scenarios of COVID-19, we selected only the SIRD model as an example to test the performance of Epi-DNNs. In addition, the data we use is official statistics, and there will be some differences from the actual data in the real world. Furthermore, the proposed Epi-DNNs method employs fully connected networks to build the model, which may not be the optimal approach. In the following works, we will try to apply other neural networks, such as RNNs and LSTMs, with the compartmental model for COVID-19 modeling.

## 6 Conclusion

In this paper, we proposed a novel Epi-DNNs method to identify the time-vary parameter for the epidemic compartmental model to accurately depict the dynamic of COVID-19. Incorporating domain knowledge, mathematical modeling, and AI techniques, we analyze the dynamics of COVID-19 using the compartment model and identify its parameters with neural networks and real-world observations. Experimental results revealed that the proposed Epi-DNNs method indeed calibrates the parameters of the compartmental model accurately and effectively. Based on the estimated parameters, reliable predictions are performed to validate the feasibility and predictability of the proposed idea to model the dynamic of COVID-19. We emphasize that our method can easily be implemented without any background knowledge about numerical analysis (for example, stability conditions) but about some libraries for implementing neural networks. Therefore, the proposed Epi-DNNs method is applicable for parameter estimation of other compartmental models and other areas around the world and future infectious diseases.

## Declaration of Competing Interest

The authors declare that they have no known competing financial interests or personal relationships that could have appeared to influence the work reported in this paper.

## Footnotes

1 https://wsjkw.sh.gov.cn/.
